# Role of lipid-mobilising factor (LMF) in protecting tumour cells from oxidative damage

**DOI:** 10.1038/sj.bjc.6601669

**Published:** 2004-02-24

**Authors:** P M Sanders, M J Tisdale

**Affiliations:** 1 Pharmaceutical Sciences Research Institute, Aston University. Birmingham B4 7ET, UK

**Keywords:** cachexia, lipid mobilizing factor (LMF), uncoupling protein-2, free radicals

## Abstract

Lipid-mobilising factor (LMF) is produced by cachexia-inducing tumours and is involved in the degradation of adipose tissue, with increased oxidation of the released fatty acids through an induction of uncoupling protein (UCP) expression. Since UCP-2 is thought to be involved in the detoxification of free radicals if LMF induced UCP-2 expression in tumour cells, it might attenuate free radical toxicity. As a model system we have used MAC13 tumour cells, which do not produce LMF. Addition of LMF caused a concentration-dependent increase in UCP-2 expression, as determined by immunoblotting. This effect was attenuated by the *β*3 antagonist SR59230A, suggesting that it was mediated through a *β*3 adrenoreceptor. Co-incubation of LMF with MAC13 cells reduced the growth-inhibitory effects of bleomycin, paraquat and hydrogen peroxide, known to be free radical generators, but not chlorambucil, an alkylating agent. There was no effect of LMF alone on cellular proliferation. These results indicate that LMF antagonises the antiproliferative effect of agents working through a free radical mechanism, and may partly explain the unresponsiveness to the chemotherapy of cachexia-inducing tumours.

Wasting of adipose tissue in cancer cachexia appears to be mediated, at least in part, by a 43 kDa glycoprotein, lipid-mobilising factor (LMF), secreted by cachexigenic tumours (Hirai *et al*, 1998), which acts to directly stimulate lipolysis through a cyclic AMP-mediated process. Lipid-mobilising factor was shown to be homologous to the plasma protein zinc-*α*2-glycoprotein (ZAG) (Todorov *et al*, 1998). In addition to stimulation of triglyceride hydrolysis, LMF also increases oxidation of the released fatty acids into carbon dioxide through increased expression of uncoupling proteins (UCPs). Lipid-mobilising factor induces increased expression of UCP-1, -2 and -3 in brown adipose tissue (BAT), and UCP-2 in both skeletal muscle and liver (Bing *et al*, 2002). UCP-1 channels protons across the inner mitochondrial membrane not linked to ATP production, and thus constitute an energy sink. While UCP-1 is found only in BAT and has a marked and strongly regulated uncoupling activity, UCP-2 is found in most tissues, but the correlation between the expression level of UCP-2 and the proton leak of the inner membrane is somewhat controversial (Cadenas *et al*, 1999; Ricquier and Bouillaud, 2000). A potential role of UCP-2 is in the detoxification of superoxide radicals (O_2_−) produced by one-electron reduction of oxygen in the mitochondria. Thus, the tumour necrosis factor *α* (TNF-*α*)-dependent increase in oxidant production by liver mitochondria caused UCP-2 induction, and this may represent an antioxidant defence mechanism (Lee *et al*, 1999). An increased reactive oxygen species (ROS) formation was also observed in hepatocytes prior to UCP-2 induction by lipid emulsion (Cortez-Pinto *et al*, 1999).

An important question is whether there is any survival advantage to tumours which produce catabolic factors such as LMF. Most tumours use glucose rather than lipids as an energy source due to the low oxygen tension, although release of polyunsaturated fatty acids, such as linoleic or arachidonic acids, may result in stimulation of tumour growth (Sauer and Dauchy, 1987). If LMF induced UCP-2 expression in the tumour, this may be important in detoxifying free radicals, which are produced in excess during the process of cachexia (Gomes-Marcondes and Tisdale, 2002; Mantovani *et al*, 2002). Many anticancer drugs such as adriamycin, bleomycin and mitomycin C exert their action through generation of reactive oxygen radicals, and induction of UCP-2 by LMF may protect tumour cells from their cytotoxic action. These agents constitute one of the most important groups of antitumour agents because they possess broad spectrum activity.

To investigate this possibility, the effect of LMF on oxidant damage induced by a range of agents was determined in a cell line (MAC13) which does not produce LMF (Todorov *et al*, 1998), and the mechanism of this effect was determined.

## MATERIALS AND METHODS

### Materials

Foetal calf serum (PCS) and RPMI 1640 tissue culture media were purchased from Life Technologies (Paisley, UK). Rabbit polyclonal antisera to mouse UCP-2 was purchased from Calbiochem through CN Biosciences UK (Nottingham, UK), while mouse monoclonal antibody to human ZAG was from Santa Cruz Biotechnology through Autogen Bio Clear (Wiltshire, UK). The secondary antibodies were obtained from Dako A/S, Denmark. All other chemicals were purchased from Sigma-Aldridge (Dorset, UK). Human ZAG and polyclonal anti-hZAG antibody were gifts from Dr T Zimmerman, Bayer Corp., Raleigh, NC, USA.

### Purification of LMF

**Figure 1:**
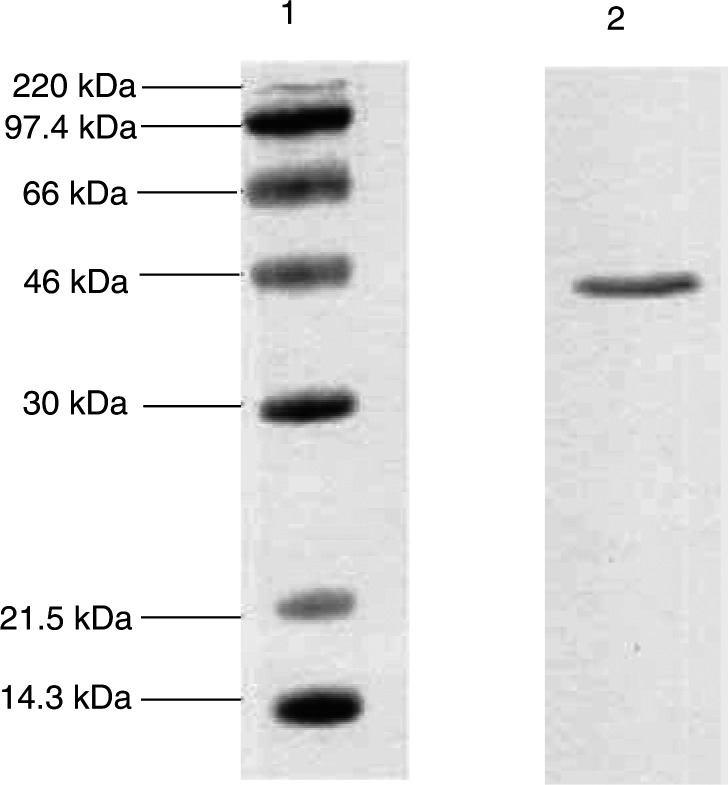
SDS–PAGE of LMF purified from human urine according to the protocol described in Materials and methods. Lane 1, MW markers; lane 2, LMF. Detection was by Coomassie brilliant blue stain.

Lipid-mobilising factor was purified from the urine of cachectic cancer patients using a combination of batch extraction on DEAE-cellulose and hydrophobic interaction chromatography (Todorov *et al*, 1998). Particulate material was removed from urine by centrifugation at 3000 × **g** for 10 min, after which it was diluted with 4 vol 10 mM Tris-HCI, pH 8.0. DEAE-cellulose, previously activated by washing in 100 mM Tris-HCI, pH 8.0, for 5 min was added to the diluted urine (10 g l^−1^ of original urine) and the mixture was stirred for 2 h at 4°C. The DEAE-cellulose was recovered by centrifugation (300 × **g**, 10 min) and the LMF was eluted with 0.5 M NaCl in 10 mM Tris-HCl, pH 8.0. The eluate was equilibrated against PBS and concentrated to 1 ml by ultrafiltration in an Amicon filtration cell (Millipore (UK) Ltd, Watford, Herts, UK) containing a membrane filtre with a molecular weight cutoff of 10 kDa. Further purification was achieved using a Resource-Iso HPLC column (Pharmacia Biotech, St Albans, Herts, UK) with a decreasing (NH_4_)_2_SO_4_ concentration from 1.5 M. Active fractions containing LMF eluted at 0.6 M (NH_4_)_2_SO_4_ and a sodium dodecylsulphate polyacrylamide (SDS–PAGE) gel showing the purity of the product is shown in Figure 1

**Figure 2:**
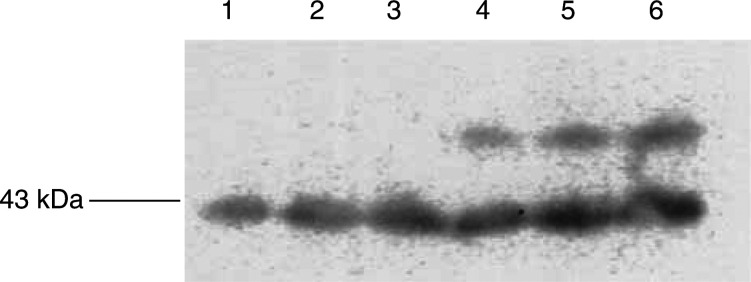
Immunoblot of hZAG (lanes 1–3) and hLMF (lanes 4–6) (10 *μ*g) detected with polyclonal antibody to hZAG.

. The LMF was present as a single band and immunoblotting showed it to be cross-reactive with human ZAG (Figure 2

). The upper band is albumin, which co-purifies with LMF to a small extent. The LMF was desalted before use by washing five times against PBS using an Amicon fitter. Zinc-*α*2-glycoprotein was kindly provided by Dr Tom Zimmerman, Bayer Corporation, USA, and was purified from Cohn Fraction V of human serum.

### Cell lines

MAC13 is a colon adenocarcinoma induced in mice by prolonged administration of 1, 2-dimethylhydrazine (Cowen *et al*, 1980). Cells were derived from the solid tumour and maintained *in vitro* in RPMI-1640 medium supplemented with 10% FCS at 37°C under an atmosphere of 5% carbon dioxide in air. For cell-growth assays, cells were taken from semiconfluent cultures and seeded at 1.6 × 10^4^ cells per well in six-well multiwell dishes (Nunc A/S Denmark) and left for 2 h before drug addition. Bleomycin, 1, 1′-dimethyl-4,4′-bipyridinium dichloride (paraquat), hydrogen peroxide or chlorambucil were then added to the cells at the concentrations shown in the figure legends in the absence or presence of 0.58 *μ*M LMF for 2 h, following which the experimental medium was discarded and replaced by fresh medium. Cell numbers were determined 96 h after drug addition using a Coulter Counter, model Z1.

### Western blot analysis

MAC13 cells were washed with ice-cold PBS, scraped from the substratum into 100 mM HEPES, pH 7.5, 10% sucrose, 0.1% NP40, 10 mM dithiothreitol and protease inhibitor cocktail (Roche Diagnostics, Germany), and sonicated at 4°C three times for 10 s, with intervals between each pulse. The protein concentration of the sample was determined using the Bradford assay using bovine serum albumin as a standard. Samples of protein (5 *μ*g in 10 *μ*l) were resolved on 12% SDS–PAGE run at 180 V for about 45 min and transferred in a Tris-glycine SDS buffer, to 0.45 *μ*m nitrocellulose membranes (Hybond A, Amersham, UK), which had been blocked with 5% Marvel in Tris-buffered saline, pH 7.5, at 4°C overnight. Both anti-UCP-2 and anti-ZAG were used at a dilution of 1 : 1000, while the secondary antibodies were peroxidase conjugated, either goat anti-rabbit or rabbit anti-mouse used at a dilution of 1 : 2000. Incubation was for 1 h at room temperature and development was by enhanced chemiluminescence (ECL) (Amersham Biosciences, Bucks, UK). Blots were scanned by a densitometer to quantitate the differences and analysed using ‘Phoretix ID Advanced’ software. Gels were stained with Ponceau S to confirm equal loading.

### Malondialdehyde (MDA) determination

Cells were homogenised in the presence of 5 mM butylated hydroxytoluene to prevent sample oxidation. Cell homogenate (0.2 ml) or 1,1,3,3-tetramethoxypropan (0.2 ml, standard) or water (0.2 ml, blank) were added to N-methyl-2-phenylindole (0.2 ml) and the mixture was vortexed prior to adding 12 N HCl (46 *μ*l) and again mixing well. The tubes were sealed and the samples incubated at 45°C for 60 min, followed by centrifugation at 15 000 × **g** for 10 min. The clear supernatant was transferred to a microtitre plate and the absorbance was measured at 570 nm using an ELISA plate reader (Athos Labtech, Sussex, UK).

### Statistical analysis

Results were expressed as mean±s.e.m. Differences were determined by one-way ANOVA followed by Tukey–Kramer multiple-comparison test.

## RESULTS

**Figure 3:**
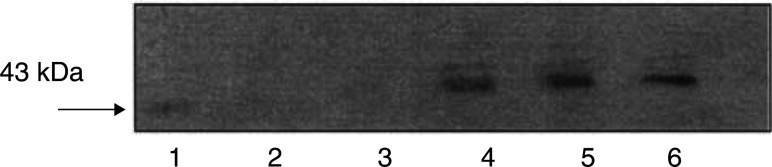
Immunoblot of soluble extracts of MAC13 (lanes 1–3) and MAC16 (lanes 4–6) detected with monoclonal antibody to human ZAG.

**Figure 4:**
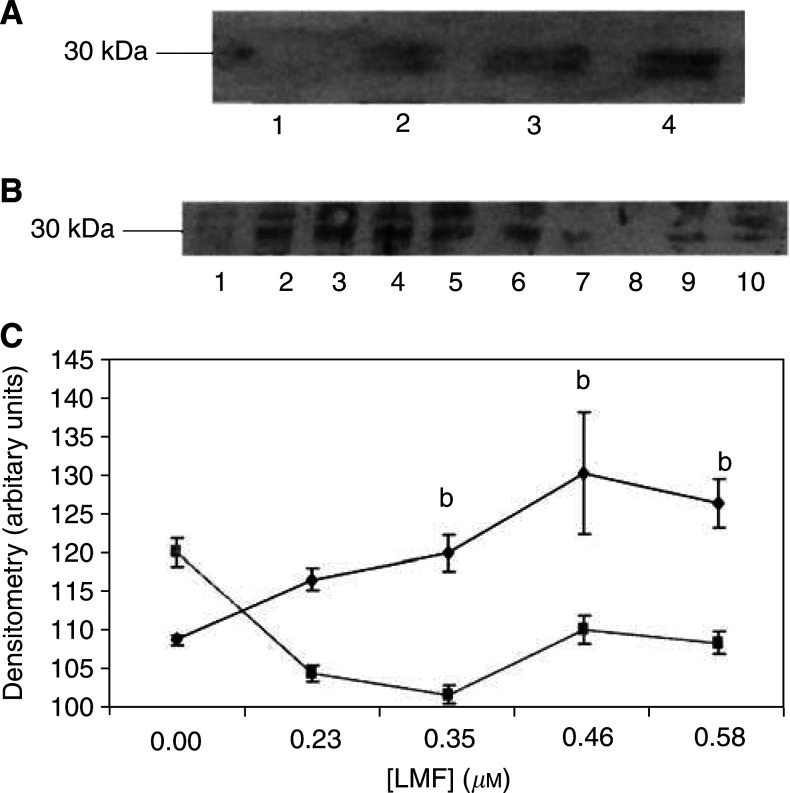
Immunoblot of UCP-2 expression in MAC13 cell line (**A**) in the presence of 0 (lane 1), 0.23 (lane 2), 0.35 (lane 3) and 0.58 (lane 4) *μ*M LMF after 24 h incubation and (**B**) in the presence of 0 (lanes 1 and 6), 0.23 (lanes 2 and 7), 0.35 (lanes 3 and 8), 0.46 (lanes 4 and 9) and 0.58 (lanes 5 and 10) *μ*M LMF for 24 h in the absence (lanes 1–5) or presence (lanes 6–10) of 10 *μ*M SR59230A. (**C**) Densitometric analysis of the blot shown in (B). The symbols ♦ are in the absence of SR59230A and ▪ in the presence; *n*=3. Differences from values in the presence of SR59230A are indicated as b, *P*<0.01.

Previous studies (Todorov *et al*, 1998) have shown LMF to be homologous to ZAG and using quantitative RT–PCR have found MAC16, which induces cachexia *in vivo*, to express high levels of ZAG mRNA, while MAC13, which does not induce cachexia, showed no expression of ZAG mRNA. Immunoblotting of soluble extracts of the two cell lines confirmed no expression of ZAG protein by MAC13, but high-level expression by MAC16 (Figure 3

). To determine the effect of LMF/ZAG on the expression of UCP-2, MAC13 cells were treated with various concentrations of LMF for 24 h and the expression of UCP-2 was determined by Western blotting. Uncoupling protein-2 appears as a doublet (Figure 4

), possibly because it acts as a dimer forming a proton channel in the mitochondrial inner membrane. As shown in Figure 4A, LMF produced a concentration-dependent increase in UCP-2, which was attenuated by co-incubation with the selective *β*3-adrenoreceptor (AR) antagonist SR59230A (Figure 4C). This suggests that the action of LMF was mediated through interaction with a *β*3-AR. ZAG produced a similar effect and appeared to be interchangeable with LMF and was used in some experiments. The concentrations of LMF inducing an increase in UCP-2 expression were the same as those previously found to induce lipolysis in murine epididymal adipocytes (Hirai *et al*, 1998). Since maximal induction of UCP-2 expression was observed with 0.58 *μ*M LMF, this concentration was chosen for further studies. There was no effect of LMF at this concentration on the growth of MAC13 cells, nor of SR59230A at 10 *μ*M.

**Figure 5:**
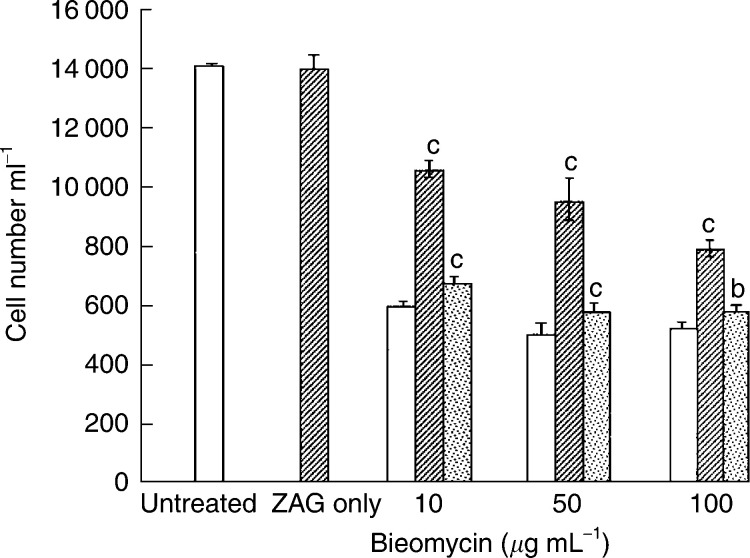
Effect of bleomycin alone on the growth of MAC13 cells (open boxes), or in the presence of 0.58 *μ*M LMF (hatched boxes), or 0.58 *μ*M LMF+10 *μ*M SR59230A (stippled boxes). Zinc-*α*2-glycoprotein alone was used at a concentration of 0.58 *μ*M. Total repeats *n*=3. Differences from values in the presence of bleomycin alone are indicated as c, *P*<0.001, white differences from bleomycin +ZAG are indicated as b, *P*<0.01, and d, *P*<0.001.

The effect of bleomycin on growth of MAC13 is shown in Figure 5

**Figure 6:**
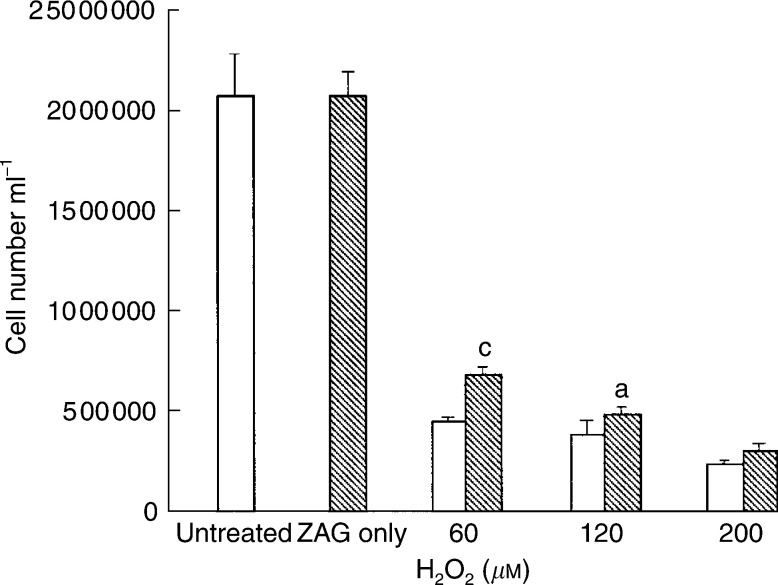
Effect of hydrogen peroxide on the growth of MAC13 cells alone (open boxes) and in the presence of 0.58 *μ*M ZAG (hatched boxes). Zinc-*α*2-glycoprotein alone was used at a concentration of 0.58 *μ*M. Total repeats *n*=3. Differences from values in the presence of hydrogen peroxide alone are indicated as a, *P*<0.05, and b, *P*<0.01.

**Figure 7:**
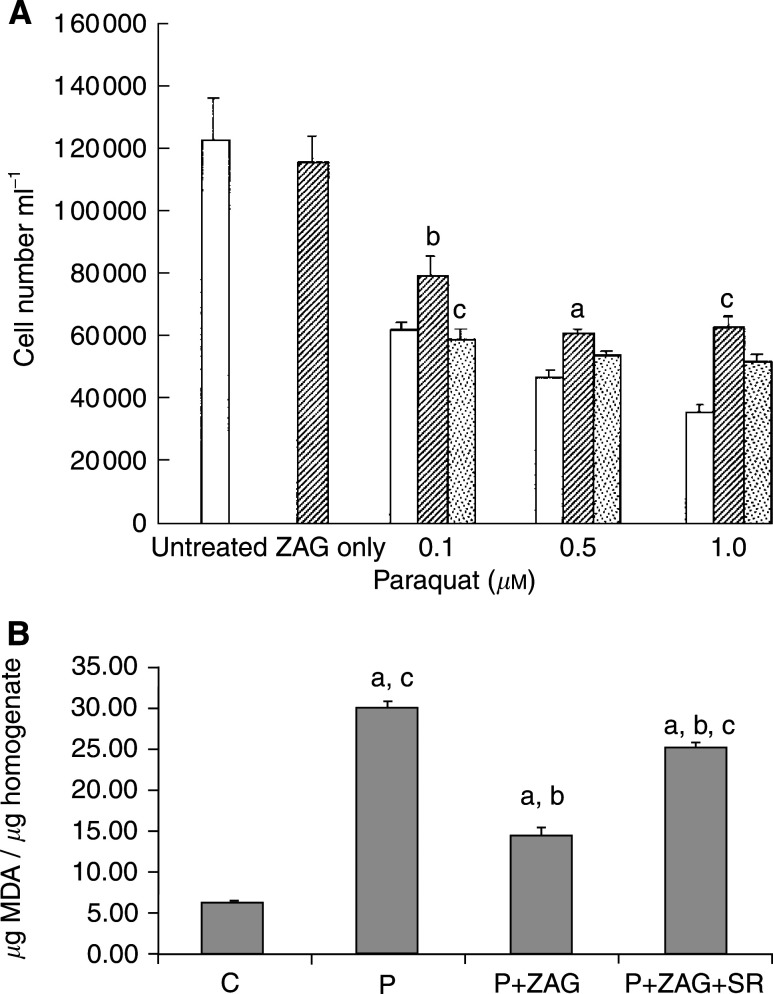
(**A**) Effect of paraquat on the growth of MAC13 cells alone (open boxes) and in the presence of 0.58 *μ*M LMF alone (hatched boxes) or with 10 *μ*M SR59230A (stippled boxes). Lipid-mobilising factor was used alone at a concentration of 0.58 *μ*M. Total repeats *n*=3. Differences from values in the presence of paraquat alone are indicated as a, *P*<0.05, and b, *P*<0.01, white differences from paraquat+LMF are indicated as c, *P*<0.001. (**B**) Levels of MDA in MAC13 cells after no treatment (**C**) or treatment with 0.1 *μ*M paraquat (P), 0.1 *μ*M paraquat+0.58 *μ*M ZAG (P+ZAG) or 0.1 *μ*M paraquat+0.58 *μ*M ZAG+10 *μ*M SR59230A (P+ZAG+SR); *n*=6. Differences from control are shown as a, *P*<0.05, while differences from 0.1 *μ*M paraquat are shown as b, *P*<0.001, and differences from P+ZAG are shown as c, *P*<0.001.

**Figure 8:**
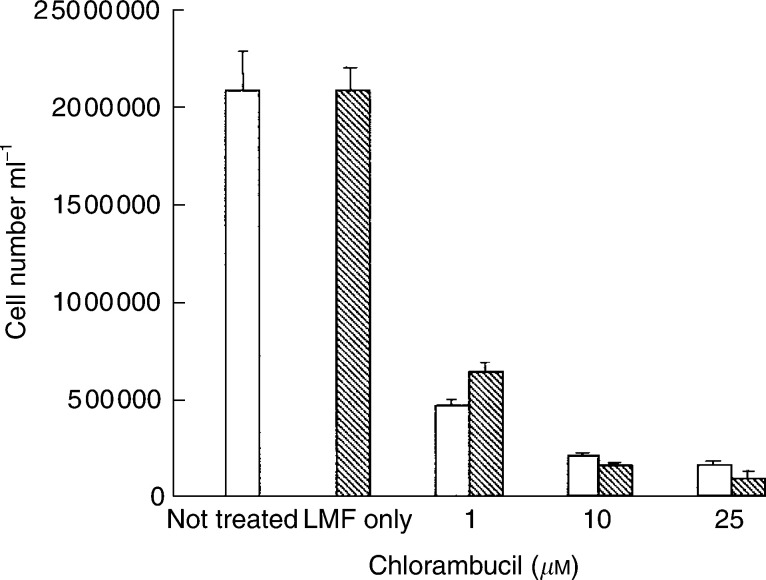
Effect of chlorambucil on the growth of MAC13 cells alone (open boxes) and in the presence of 0.58 *μ*M LMF (hatched boxes). Lipid-mobilising factor was used alone at a concentration of 0.58 *μ*M. There were no significant differences between the two groups.

. There was a significant reversal of growth inhibition by bleomycin at all concentrations when cells were co-incubated with ZAG (0.58 *μ*M), and this growth-enhancing effect was completely attenuated in the presence of SR59230A, suggesting that the protective effect of ZAG was mediated through *β*3-AR and thus UCP-2 (Figure 5). A similar protective effect was seen by ZAG on the growth-inhibitory effect of hydrogen peroxide (Figure 6

), although the protective effect decreased as the concentration of hydrogen peroxide increased and was not apparent in the presence of 200 *μ*M hydrogen peroxide. Another free-radical generator is paraquat, which promotes a flux of O_2_− within cells by transferring single electrons catalytically from biological reductants and is toxic to cells (Haly, 1979). Paraquat produced a dose-dependent decrease in proliferation of MAC16 cells, which was effectively attenuated in the presence of LMF (Figure 7A

). There was a significant increase of MDA, a recognised marker of oxidative stress, in the presence of paraquat, and this was significantly attenuated in the presence of 0.58 *μ*M ZAG (Figure 7B). The suppressive effect of ZAG on MDA production in the presence of paraquat was reversed by SR59230A, confirming that the action of ZAG was mediated through the *β*3-AR. In contrast to the action of agents inducing oxidative stress, chlorambucil, an alkylating agent which inhibits cellular proliferation by cross-linking DNA (Harrap and Gascoigne, 1976), inhibited the growth of MAC13 cells (Figure 8

), but this growth inhibition was not attenuated by LMF.

## DISCUSSION

Weight loss is a common feature in patients with gastrointestinal tumours, and is an independent variable of outcome considerably reducing survival (DeWys, 1985). Weight loss in these patients decreases the response to chemotherapy, possibly because they receive lower dose levels because of increased toxicity (Andreyev *et al*, 1998). Weight loss is most likely to have an impact on response when the rate of response is in the 40–80% range (DeWys, 1985). One potential mechanism for the decreased response to chemotherapy is that cachectic factors elaborated by tumours, and producing lipolysis in adipose tissue or protein degradation in skeletal muscle, may interfere with the action of anticancer drugs. This study has shown that one such factor, LMF, has the potential to decrease growth inhibition by agents inducing free radical damage through an increased expression of UCP-2. Further support for this suggestion is provided by the high chemosensitivity of the MAC13 tumour, which does not produce LMF, and the chemoresistance of the MAC16 tumour, which secretes LMF (Double and Bibby, 1989). Lipid-mobilising factor is produced only by cachexia-inducing tumours and is immunologically identical to ZAG. ZAG is a soluble protein first isolated from human plasma (Bürgi and Schmid, 1961), the name of which derives from the tendency to precipitate with zinc salts and its electrophoretic mobility in the regions of the *α*2-globulins. Although ZAG was isolated over 40 years ago, the biological function remained largely unknown until it was discovered to be homologous with LMF involved in lipid mobilisation in cancer cachexia (Hirai *et al*, 1998). Stimulation of lipolysis by LMF occurs through a cyclic AMP-dependent process, which appears to be mediated through a *β*3-AR (Russell *et al*, 2002).

Induction of UCP-2 expression in MAC13 cells by LMF was attenuated by the *β*3-AR antagonist SR59230A (Nisoli *et al*, 1996), suggesting that the *β*3-AR was involved in the upregulation. Up regulation of UCP-2 in liver by polyunsaturated fatty acids appears to involve a prostaglandin/peroxisome-proliferator-activated receptor *α* (PPAR*α*)-mediated pathway (Armstrong and Towle, 2001), while thiazolidinediones appear to work through a PPAR*α* pathway in muscle and adipose tissue (Camirand *et al*, 1998). In addition, a cyclic AMP-response element has been found in the mouse UCP-2 promoter region and increased mRNA for UCP-2 was found in 3T3-L1 adipocytes in the presence of cyclic AMP analogues (Yoshitomi *et al*, 1999). In skeletal muscle, catecholamines upregulate both UCP-2 and UCP-3 mRNA expression through direct action on the *β*2-AR (Nagase *et al*, 2001). The *β*3-AR agonist Tertatol increased UCP-2 expression in white adipose tissue (Margarets *et al*, 2001), but this was probably due to fatty acid release from adipose tissue, since UCP-2 is also regulated by the fat content on the diet (Fleury *et al*, 1997). However, the effect of LMF was exerted on MAC13 tumour cells in the absence of fatty acids, suggesting a direct effect through the *β*3-AR. The mechanism of this effect is not known, but most likely involves a cyclic AMP-mediated process.

If UCP-2 is involved in the reduction of free radical formation (Ricquier and Bouillaud, 2000), then it was predicted that it would antagonise, the antiproliferative effects of agents acting through a free radical mechanism. Bleomycin is an antitumour antibiotic which degrades DMA in a process dependent on the chelation of ferrous ions, which under aerobic conditions leads to the formation of the hydroxyl radical in a concerted process, which is thought not to involve free hydroxyl radicals (Favaudon, 1982). Despite this, LMF attenuated the antiproliferative effect of bleomycin in a process which possibly involves the *β*3-AR, since it was completely reversed in the presence of SR59230A. The antiproliferative effect of paraquat and hydrogen peroxide was also antagonised by LMF, but not that of the alkylating agent chlorambucil. In addition, production of MDA by paraquat, a recognised marker of oxidative stress, was shown to be significantly reduced by ZAG, and the ZAG effect was attenuated by SR59230A, confirming that it arose through the *β*3-AR. These results suggest that one reason for the poor response of cachexia-inducing tumours to chemotherapy may be the increased expression of UCP-2 and detoxification of free radical-generating agents by tumour-produced LMF.
